# Co-Occurrence of Health Conditions during Childhood: Longitudinal Findings from the UK Millennium Cohort Study (MCS)

**DOI:** 10.1371/journal.pone.0156868

**Published:** 2016-06-09

**Authors:** Kathryn R. Hesketh, Catherine Law, Helen Bedford, Steven Hope

**Affiliations:** UCL Institute of Child Health, Guilford Street, London, United Kingdom; University of Stellenbosch, SOUTH AFRICA

## Abstract

**Aims:**

To identify patterns of stability and change in co-occurrence in children between 5–11 years, and to assess if they vary by socio-demographic factors.

**Methods:**

Data from 9548 singleton children from the UK Millennium Cohort Study (MCS) were assessed for co-occurrence of five common adverse conditions: wheeze; longstanding illness; unfavorable weight; injury; and socio-emotional difficulties. We summed adverse conditions (0–5) for each child at ages 5, 7, and 11 and identified co-occurrence (≥2 conditions). Using multinomial regression, we explored associations between co-occurrence trajectories and child’s sex and ethnicity, maternal education, and income quintile.

**Results:**

45.6% of children experienced co-occurrence between 5–11 years (7% experienced constant co-occurrence). More children moved into co-occurrence than moved out (16.9 vs. 11.9%). Mutually-adjusted relative risk ratios (aRRR) showed a gradient by maternal education: compared to children with no co-occurrence whose mothers had a higher/degree, children whose mothers had no qualifications were more likely to move into (aRRR = 1.32(95%CI:1.02,1.70)), out of (1.74(1.34,2.26)), have fluctuating (1.52(1.09,2.10)) or constant co-occurrence (2.58(1.76,3.80)). The same gradient (high vs. low) was evident for income quintiles. Girls were less likely to experience co-occurrence.

**Conclusions:**

Co-occurrence of adverse conditions is common during childhood, and trajectories are socially patterned. Child-focused care for lower-income children and boys early in life may prevent and reduce co-occurrence in later childhood.

## Introduction

Poor health in childhood may be harmful to children’s social and academic development,[1,2] and predicts poor health in adulthood.[3–5] Experiencing multiple adverse health conditions as a child may therefore add to this burden, leading to adverse health in adulthood that is worse than that resulting from single conditions. However, relatively little is known about co-occurrence of health conditions (i.e. having two or more adverse conditions) in childhood and whether the burden of co-occurrence changes with increasing age. Both are important for public health provision, as co-occurrence is likely to have consequences for a child’s current and future health and development,[6] and for health care provision.[7]

Studies exploring cross-sectional relationships between two or three health conditions report that obesity and emotional wellbeing,[8] and various allergy-related conditions[9,10] frequently occur together. To our knowledge, only two studies to date have assessed how multiple health conditions co-occur in childhood,[11,12] with contrasting results. One study, assessing multiple physical and mental adverse conditions (e.g. stroke, kidney or vascular disease, epilepsy), reported a low prevalence (1.9%) of co-occurrence in Scottish children and young adults (0–24 years).[12] In contrast, when conditions relevant to children were assessed (e.g. asthma, ability to pay attention or learn), co-occurrence of adverse physical health, emotional and developmental outcomes was identified in almost half (49.5%) of US four-year-old children.[11] This latter study also found that co-occurrence differed by gender and socio-economic circumstances, with boys and children from lower income homes at greater risk of experiencing co-occurring adverse health conditions.[11]

Multiple adverse health conditions in childhood may therefore present a particular burden for disadvantaged families and boys. Importantly, co-occurrence appears to be common in early[11] childhood, but the extent to which this may be influenced by a child’s socio-demographic circumstances remains unknown. Socio-demographic inequalities in individual health conditions have been shown to exist during childhood,[13] though evidence suggests that ‘youth’ (i.e. (early) adolescence)), may be characterized by relative health equity.[14] Reductions in inequalities as children age is apparent for several major single health conditions (e.g. acute illness, accidents and injuries, and mental health) with the exception of severe chronic illness.[14]

This ‘equalization’ is suggested to arise when the influences of the family and home environment diminish, with (secondary) school, peers and youth culture playing a larger role in children’s lives.[14] Moreover, promoting optimal child health, particularly through early intervention, has been a public health priority for successive governments in the UK since 1997.[15–17] Given implementation of multiple policy interventions to improve health, combined with a tendency towards health equalization among children moving from later childhood into adolescence, one may expect that the burden of adverse health conditions in UK children should therefore be stable or decreasing.

Using a population-based sample of UK children born between 2000–02, we therefore sought to explore whether the burden of co-occurrence in children changed between the ages of 5 and 11, and if changes differed by socio-demographic factors.

## Methods

### Subjects and design

We used data from the Millennium Cohort Study (MCS), a longitudinal study of children born in the UK between September 2000 and January 2002 which has been described elsewhere.[18] Briefly, the first contact with the cohort child was at age 9 months, with survey interviews carried out by trained interviewers in the home with the main respondent (usually the mother) and their partner, where present. A disproportionally stratified clustered sampling design was used to over-represent children living in Wales, Scotland and Northern Ireland, disadvantaged areas and areas with high proportions of ethnic minority groups.[19] Information was collected on 72% of those approached, resulting in an initial sample of 18, 552 infants (18, 296 singletons). Data were collected at four further sweeps when the children were aged 3, 5, 7, and 11 years old.[18] This paper uses data from the 9548 singleton children who participated in the 5, 7 and 11 year sweeps (MCS3-5), with complete case data for the five adverse health conditions and socio-demographic characteristics. Data were obtained from the UK Data Service, University of Essex in March 2014. Ethical approval was granted for all sweeps of the MCS, and for the fifth sweep was granted by the Yorkshire and Humber Research Ethics Committee in July 2011 (Ref: 11/YH/0203).

### Measures

#### Health conditions

The Child and Youth Health conditions framework (CYPHOF) [20] and Children and Young People’s Health Benchmarking tool[7] were developed in the UK as a resource for health and social care professionals to monitor children’s health and social care outcomes, and to improve services (and subsequently outcomes).[7,21,22] The benchmarking tool sets out a range of health and social outcomes which can be measured as part of service delivery, and is relevant from birth to 18 years.[7,20] The conditions included in the co-occurrence index were guided by CYPHOF outcomes that were available in the MCS and that are prevalent in the sample. In addition, we used responses to questions about parent-reported limiting illness to identify burden of ill-health from conditions that would not have been identified otherwise. We included five common adverse physical and mental health conditions in analyses, where ‘common’ is defined as ≥10% prevalence in the sample. Physical health indicators included wheeze, injury requiring health service attendance, longstanding illness (excluding those measured in other included conditions), and unfavorable weight (including thinness, overweight and obesity). Mental health was measured with the Strengths and Difficulties Questionnaire (SDQ).[23,24] Using the emotional, peer, conduct disorder and hyperactivity subscales, we derived a total SDQ score which was dichotomized using the standard cut-off for borderline/ abnormal socio-emotional behavior (see Table 1 for the derivation of health conditions).

**Table 1:** Measures of adverse health conditions, their derivation and prevalence at age 5, 7 and 11 years (n = 9548).

Measure	Method of measurement	Variable label (coding)	Weighted % (n) prevalence of adverse condition (1)		
			Age 5	Age 7	Age 11
**Wheeze**	Main respondent report whether child had wheezing or whistling in the chest in the last 12 months.	Yes (1) or no (0)	17.4 (1636)	13.7 (1281)	11.8 (1132)
**Longstanding illness**	Main respondent report that child has a current longstanding illness (e.g. epilepsy, hearing or sight conditions, cancer, digestive or circulation problems). Conditions measured in separate indicators (wheeze, injury, overweight/obesity and conduct /emotional/social disorders) were excluded.	Yes (1) or no (0)	14.5 (1371)	7.5 (680)	13.3 (1256)
**Injury**	Main respondent report of an unintentional injury for which medical attention was sought between sweep (e.g. in the time between MCS sweep 2 and sweep 3 for Age 5 data).	Yes (1) or no (0)	28.2 (2616)	23.7 (2210)	37.9 (3517)
**BMI**	BMI (Weight/Height^2^)—derived from heights and weights measured by trained interviewers. BMI z-score standardised, and classified according to the published cut-offs for thinness[25] (≤grade 1), and normal range BMI, overweight (excluding obesity), and obesity[26]	Unfavourable weight (i.e. underweight, overweight or obese) (1) or Healthy BMI (0)	24.3 (2363)	24.5 (2378)	32.8 (3164)
**Total SDQ score**	Main respondent completed the SDQ questionnaire about the child’s current socio-emotional state at each sweep. Sum of ‘emotional’, ‘peer’, ‘conduct’ and ‘hyperactivity’ SDQ subscales. Dichotomised based on published recommendations.[24]	Borderline/ Abnormal (≥14; 1) or Normal (0–13; 0)	10.8 (965)	12.7 (1117)	14.9 (1303)

MCS: Millennium Cohort Study; BMI: Body Mass Index; SDQ: Strengths and difficulties questionnaire.

#### Socio-demographic characteristics

Four socio-demographic variables were included in analyses. Child’s sex and ethnic group, highest level of maternal education and quintiles of household income (calculated using a modified OECD equivalence scale) were obtained when the children were 9 months old. These latter two socio-economic variables were chosen to represent children’s early life influences, though we also tested whether results were similar using socio-economic indicators recording at later sweeps (see below).

### Analysis

We first derived a co-occurrence index by summing the total number of adverse health conditions a child had at each of the three time points (0–5; at ages 5, 7 and 11) (S1 Table). As we aimed to assess the burden of ill-health, each condition was weighted equally in the index, as has been done previously;[12] although we acknowledge that the severity of conditions may have differed by child, we did not have sufficient information about the severity of individual children’s conditions to derive a weighted index.

Using the co-occurrence index, we created a binary variable at each age to categorize health burden. Preliminary analyses indicated that children with 0 or 1 adverse health condition did not differ significantly by sex, ethnicity or socio-economic variables. Children with no or one outcome were therefore coded 0, whilst children experiencing two or more adverse health conditions were coded 1 (‘co-occurrence’). Combining those with ‘co-occurrence’ (i.e. 2 or more conditions[27]), allowed us to identify children with a greater burden of ill-health. Although it would have been preferable to retain the ordinal variable (0–5 conditions) for analyses, it was not practical to do this, as this would have resulted in 216 different possible trajectories (6^3^).

The cross-sectional binary markers of co-occurrence were used to generate five co-occurrence trajectories (Table 2): 1) **no co-occurrence,** 2) **into** co-occurrence, 3) **out of** co-occurrence, 4) **fluctuating** co-occurrence and 5) **constant** co-occurrence. Generating these trajectories allowed us to explore the equalization hypothesis in more details, comparing prevalence of children moving into and out of co-occurrence between 5–11 years. Specifically, we compared how the risk of children moving into, out of, having fluctuating and constant co-occurrence between 5 and 11 years differed from those with no co-occurrence. We then assessed whether these trajectories were influenced by children’s socio-demographic factors and tested for possible effect modification.

**Table 2:** Classification of co-occurrence trajectories from age 5 to 11 (n = 9548)

	Age 5	Age 7	Age 11	% Prevalence (n)	
**No co-occurrence**	0	0	0	54.4 (5194)	**54.4 (5194)**
**Into co-occurrence**	**0**	**1**	**1**	4.4 (420)	**16.9 (1614)**
	**0**	**0**	**1**	12.5 (1194)	
**Out of co-occurrence**	**1**	**0**	**0**	8.8 (840)	**11.9 (1136)**
	**1**	**1**	**0**	3.1 (296)	
**Fluctuating co-occurrence**	**1**	**0**	**1**	5.4 (516)	**9.8 (936)**
	**0**	**1**	**0**	4.4 (420)	
**Constant co-occurrence**	**1**	**1**	**1**	7.0 (668)	**7.0 (668)**

0 represents child with 0/1 adverse health outcome at sweep;

1 represents child with 2 or more adverse health conditions at sweep.

We used multinomial regression analyses to estimate relative risk ratios (RRR) for these trajectories, using the no co-occurrence group as the baseline comparator. A RRR of less than one therefore indicates that risk is higher in reference trajectory, and when greater than one, the risk is higher in the comparison trajectory. We first conducted univariable analyses adjusting for sex, ethnic group, highest level of maternal education and quintile of household income when the cohort child was 9 months. We then conducted multivariable analyses, adjusting for all four socio-demographic variables simultaneously. As no effect modification by sex, maternal educational attainment, household income or ethnicity was apparent, we analyzed the sample as a whole.

Analyses were conducted using STATA 13/SE;[28] sample weights were used to account for the MCS complex survey design and cohort attrition over time. This weight takes into account factors such as the stratum children were recruited from (dis/advantaged in England, Wales, Scotland and Northern Ireland and ethnicity in England), and also cohort attrition up until and including the 5^th^ sweep (at age 11).[19]

We conducted a number of sensitivity analyses. We tested whether the results obtained using measures of socioeconomic circumstances (household income and maternal education) at nine months differed when the equivalent measures recorded at ages 5 and 11 years were used. We also assessed whether results differed according to the age at which a child moved into or out of co-occurrence (i.e. at 7 vs. 11 years old). Finally, we analyzed the effect of data missingness, repeating the analyses using multiply imputed data,[29] to examine whether limiting our sample to complete cases biased our findings. We used the ‘mi impute chained’ command in Stata to impute the five health conditions used to derive the co-occurrence index at each sweep. We conducted two imputation analyses: 1) using only exposure data to impute the data and 2) additionally including available data from previous/ subsequent sweeps for the health conditions (e.g. for the sweep at age 7, we included data from age 5 and 11 to impute outcome variables). As relatively little data were missing for early sweeps, we used the following exposure variables to impute the datasets: sex; ethnicity; maternal educational attainment at 9 months; quintiles of household income at 9 months; maternal age at birth; birth weight; duration of breastfeeding; number of children and total number of people in the household; language spoken at home and stratum from which the cohort family were sampled (i.e. England: high/low deprivation or ethnic). We imputed 10 datasets per sensitivity analysis, which we used to re-run our original analyses using the ‘mi svyset’ command.

## Results

Prevalences of the five adverse health conditions varied between ages 5, 7 and 11 (Table 1), with no consistent pattern in the prevalence of individual conditions. Between ages 5 and 11, the majority of children did not experience co-occurrence (54.4%); a greater proportion of children moved into co-occurrence than out between 5 and 11-years-old (16.9 vs. 11.9%) (Table 2). At age 5, boys, children of mothers with lower educational attainment and those from lower income homes were more likely to have co-occurrence (Table 3). The prevalence of co-occurrence was also higher in each of these groups at age 7 and 11, though there did not appear to be a pattern by child’s ethnicity. Trajectories between 5–11 years comprised 64 different combinations of conditions; co-occurrence at age 5, 7 and 11 was composed of 32 different combinations of the five conditions (S2 Table).

**Table 3:** Prevalence of co-occurrence at ages 5, 7 and 11 by socio-demographic factors (n = 9548).

	Age 5		Age 7		Age 11	
	No co-occurrence	Co-occurrence	No co-occurrence	Co-occurrence	No co-occurrence	Co-occurrence
Prevalence (% (n))	75.1 (7171)	24.9 (2377)	80.3 (7667)	19.7 (1881)	70.0 (6684)	30.0 (3864)
**Sex**						
Male	48.9 (3482)	57.0 (1291)	49.4 (3779)	56.9 (994)	48.8 (3267)	55.7 (1506)
Female	51.1 (3755)	43.0 (1020)	50.6 (3973)	42.7 (802)	51.2 (3507)	44.3 (1268)
**Ethnicity**						
White	92.0 (6460)	89.3 (1994)	91.7 (6909)	89.7 (1545)	909 (5963)	92.2 (2491)
Mixed	2.3 (176)	3.0 (63)	2.4 (184)	2.9 (55)	2.6 (174)	2.2 (65)
Indian	1.3 (149)	1.6 (53)	1.3 (155)	1.7 (47)	1.4 (155)	1.4 (47)
Pakistani/ Bangladeshi	2.0 (243)	2.7 (115)	2.0 (273)	2.7 (85)	2.2 (260)	1.9 (98)
Black	1.8 (150)	2.6 (63)	1.9 (168)	2.3 (45)	2.1 (158)	1.8 (55)
Other	0.7 (59)	0.7 (23)	0.7 (63)	0.8 (19)	0.1 (64)	0.5 (18)
**Maternal Educational attainment (9 months)**						
Higher/degree	19.8 (1611)	13.3 (370)	19.8 (1730)	11.6 (251)	20.0 (1527)	14.0 (454)
Diploma	9.9 (766)	7.3 (188)	9.8 (802)	7.4 152)	9.8 (704)	8.0 (250)
A-levels	10.9 (817)	8.6 (216)	10.9 (869)	8.3 (164)	10.9 (769)	9.0 (264)
GCSEs	46.0 (3101)	51.1 (1114)	46.5 (3327)	51.6 (888)	45.7 (2863)	50.7 (1352)
None	13.4 (800)	19.6 (381)	13.4 (870)	21.1 (311)	13.5 (774)	18.3 (407)
**Household Income (9 months)**						
Q1 (high)	20.9 (1618)	13.9 (357)	20.8 (1724)	12.4 (251)	21.1 (1526)	14.7 (449)
Q2	21.6 (1624)	16.8 (435)	21.7 (1748)	15.0 (311)	21.7 (1528)	17.3 (531)
Q3	20.6 (1494)	20.0 (458)	20.6 (1593)	20.0 (359)	20.7 (1403)	20.0 (549)
Q4	19.2 (1360)	22.0 (499)	19.1 (1452)	23.2 (407)	18.7 (1243)	22.7 (616)
Q5 (low)	17.6 (1141)	27.4 (562)	17.8 (1235)	29.5 (468)	17.8 (1074)	25.3 (629)

A-level: Advanced level qualification (equivalent to high school graduation (Age 18)); GCSEs: General Certificate of Secondary Education (taken at the end of compulsory education (age 16)).

In unadjusted analyses, boys (vs. girls), children of mothers with lower educational attainment (vs. those with a higher/degree) and children from lower income homes (vs. those in the highest income quintile) were at a greatest risk of all forms of co-occurrence (i.e. experiencing any trajectory). These children were more likely to move into, move out of, have fluctuating, and constant co-occurrence. Although there was a general trend for non-white children to move out of co-occurrence, due to small numbers of children in several ethnic groups, not all of these results reached statistical significance.

The elevated risks for boys and children from more disadvantaged homes remained in models mutually adjusted for socio-demographic variables (Table 4; risks can be compared both down (within socio-demographic variables) and also across (co-occurrence trajectories)). For example, for children of mothers with no educational qualifications (vs. those with higher/degree), the adjusted relative risk ratio (aRRR) ranged from 1.32 (95% CI: 1.02, 1.70) for moving into co-occurrence to 2.58 (1.76, 3.80) for having constant co-occurrence. Risks were also higher for children in the lowest income quintiles (vs. those in the highest income quintiles); the aRRR of moving into co-occurrence between age 5 and 11 was 1.67 (1.33, 2.11), and 3.18 (2.28, 4.45) for having constant co-occurrence.

**Table 4:** Associations between co-occurrence trajectories (5–11 years) and socio-demographic circumstances (n = 9548).

	No co-occurrence	Into co-occurrence		Out of co-occurrence		Fluctuating co-occurrence		Constant co-occurrence	
		RRR (95% CI)							
		Unadjusted	Adjusted	Unadjusted	Adjusted	Unadjusted	Adjusted	Unadjusted	Adjusted
**Sex** (ref: Male)									
Female	1.00	**0.85 (0.75, 0.96)**	**0.84 (0.74, 0.95)**	**0.80 (0.70, 0.91)**	**0.79 (0.69, 0.90)**	**0.68 (0.59, 0.79)**	**0.67 (0.58, 0.78)**	**0.59 (0.48, 0.71)**	**0.57 (0.47, 0.69)**
**Ethnicity** (ref: White)									
Mixed	1.00	1.00 (0.66, 1.51)	0.93 (0.62, 1.40)	**1.94 (1.23, 3.04)**	**1.82 (1.18, 2.82)**	0.91 (0.53, 1.54)	0.85 (0.50, 1.44)	1.03 (0.60, 1.76)	0.86 (0.51, 1.49)
Indian	1.00	0.82 (0.45, 1.48)	0.83 (0.45, 1.54)	1.47 (0.91, 2.39)	1.52 (0.92, 2.50)	1.11 (0.65, 1.93)	1.12 (0.63, 2.00)	1.55 (0.53, 4.54)	1.63 (0.59, 4.50)
Pakistani/ Bangladeshi	1.00	1.14 (0.78, 1.65)	0.94 (0.64, 1.38)	**2.15 (1.54, 2.99)**	**1.78 (1.26, 2.51)**	1.49 (0.94, 2.35)	1.18 (0.74, 1.87)	1.21 (0.75, 1.94)	0.81 (0.49, 1.33)
Black	1.00	0.79 (0.46, 1.34)	0.71 (0.40, 1.28)	**1.82 (1.08, 3.06)**	1.65 (0.98, 2.76)	1.51 (0.80, 2.88)	1.32 (0.69, 2.51)	1.03 (0.47, 2.29)	0.82 (0.35, 1.90)
Other	1.00	0.72 (0.31, 1.66)	0.71 (0.30, 1.69)	1.24 (0.53, 2.94)	1.27 (0.51, 3.17)	0.52 (0.20, 1.38)	0.52 (0.19, 1.40)	0.92 (0.28, 3.08)	0.94 (0.26, 3.36)
Maternal Educational attainment* (ref: Higher/degree)									
Diploma	1.00	1.10 (0.87, 1.37)	1.02 (0.81, 1.29)	0.99 (0.75, 1.31)	0.93 (0.70, 1.24)	**1.46 (1.11, 1.94)**	1.36 (1.01, 1.83)	**1.58 (1.06, 2.35)**	1.38 (0.92, 2.07)
A-levels	1.00	1.07 (0.86, 1.35)	0.97 (0.77, 1.22)	1.05 (0.80, 1.37)	0.96 (0.73, 1.27)	**1.38 (1.05, 1.82)**	1.23 (0.91, 1.67)	**1.50 (1.03, 2.18)**	1.20 (0.81, 1.76)
GCSEs	1.00	**1.43 (1.22, 1.68)**	1.18 (0.99, 1.42)	**1.52 (1.26, 1.84)**	**1.31 (1.05, 1.62)**	**1.52 (1.26, 1.84)**	1.29 (1.00, 1.66)	**3.25 (2.47, 4.27)**	**2.13 (1.57, 2.89)**
None	1.00	**1.77 (1.41, 2.21)**	**1.32 (1.02, 1.70)**	**2.08 (1.62, 2.67)**	**1.74 (1.34, 2.26)**	**2.08 (1.62, 2.67)**	**1.52 (1.09, 2.10)**	**5.06 (3.59, 7.13)**	**2.58 (1.76, 3.80)**
Household income* (ref: Q1 High)									
Q2	1.00	1.18 (0.97, 1.44)	1.14 (0.93, 1.40)	**1.31 (1.07, 1.60)**	**1.26 (1.03, 1.56)**	1.01 (0.80, 1.28)	0.96 (0.75, 1.22)	1.31 (0.92, 1.87)	1.13 (0.79, 1.62)
Q3	1.00	**1.29 (1.05, 1.59)**	1.20 (0.96, 1.51)	**1.45 (1.17, 1.79)**	**1.29 (1.02, 1.62)**	**1.36 (1.04, 1.77)**	1.23 (0.92, 1.67)	**2.17 (1.57, 2.98)**	**1.62 (1.16, 2.26)**
Q4	1.00	**1.63 (1.33, 2.01)**	**1.47 (1.16, 1.86)**	**1.67 (1.34, 2.08)**	**1.36 (1.06, 1.74)**	**1.72 (1.33, 2.23)**	**1.51 (1.13, 2.02)**	**3.13 (2.29, 4.26)**	**2.14 (1.53, 2.98)**
Q5 (low)	1.00	**1.90 (1.54, 2.34)**	**1.67 (1.33, 2.11)**	**2.31 (1.84, 2.89)**	**1.74 (1.34, 2.26)**	**2.04 (1.56, 2.67)**	**1.74 (1.28, 2.37)**	**4.92 (3.65, 6.63)**	**3.18 (2.28, 4.45)**

RRR: relative risk ratio; Adjusted analyses adjusted for all other variables in the table; bold indicates significant result *p*<0.05;

* Measured at 9 months.

Results of the sensitivity analyses showed that the associations between co-occurrence trajectories and maternal education status or quintile of household income were similar regardless of whether socio-economic variables were measured at 9 months, at age 5 or 11 years. The results of the trajectory analysis were also similar whether children moved into or out of co-occurrence at age 7 or at age 11 (analyses not shown). Multiple imputation analyses showed the same pattern of results as those conducted using complete cases (S3 Table). Children included in analyses here (compared to those excluded) were more likely to have better educated mothers (p<0.001); be from higher income homes (p<0.001); be heavier at birth (mean z score: -0.04 (SE: 0.01) vs. -0.15 (0.01)) and be white (vs. non-white, p<0.001). There were no differences by child’s sex.

## Discussion

Almost half of children in this sample experienced co-occurrence between the ages of 5–11: more children moved into co-occurrence than out, and 7% of children had constant co-occurrence (i.e. at all three time points). Both cross-sectionally and longitudinally, co-occurrence was socially patterned; compared to children with no co-occurrence between ages 5 and 11, those who experienced co-occurrence (i.e. move into, out of, have fluctuating and constant co-occurrence) were more likely to be boys, have mothers with lower educational attainment, and be from lower income homes. Compared to white children, certain groups of non-white children appeared to be more likely to move out of co-occurrence over time. These inequalities in co-occurrence exited regardless of the age at which socio-economic indicators were measured (e.g. at 9 months, 5 or 11 years), and when children moved into and out of co-occurrence (i.e. at age 7 or 11).

### Methodological considerations

To our knowledge, no other study to date has assessed how co-occurrence of multiple adverse health conditions in children changes over time, with studies tending to explore only one or two health conditions at a time,[8–10] and using cross-sectional data.[11,12] We used a large population-based sample of UK children to explore how co-occurrence of multiple adverse health conditions changed between the ages of 5 and 11. We considered a range of adverse health conditions identified as important in children,[7] and included both physical and mental health conditions, given their mutual importance for children’s wellbeing.^1^ We were however constrained by the available data, which meant we were only able to assess common health conditions reported in the study, and periods of time between data collection sweeps varied. Weight status was assessed using objective measures of height and weight at each time point, though the remaining physical and mental health conditions were assessed by main respondent report, generally the child’s mother. Parental report of child’s long-standing illness has been shown to accurately reflect recorded illness and child’s self-reported illness,[30] and maternal report of longstanding illness has a medium to high level of agreement with medical records.[31,32] As asthma phenotypes vary,[33] we used presence of wheeze in the preceding 12 months as an outcome, which may be indicative of persistent wheeze and/or asthma.[34]

The results presented here are based on complete case analysis. As tends to be the case in longitudinal cohort studies, remaining children included here were more likely to have mothers with higher educational attainment, come from higher income homes and be White. However, we used weighting to account for attrition in our analyses, and, using multiply imputed data to account for missingness, we showed results were very similar, indicating the robustness of our reported findings. We used a relatively simple measure of co-occurrence (an index) though there were many different combinations of adverse health conditions cross-sectionally and longitudinally. We also combined children with no or one health outcome, and those with two or more conditions, at each sweep as we sought to assess the difference between those with and without co-occurrence. In doing so, we may have masked the complexity between these different groups of children. Moreover, the adverse health conditions resulting in a classification of co-occurrence may have changed in some children over the measurement period while remaining the same in others. The trajectories are therefore a simplification of children’s patterns of co-occurring conditions over time. However, they provide an indication of the prevalence of multiple adverse health conditions and how these change during childhood, and whether this varies by a child’s socio-demographic characteristics. We considered other methods of analysis, which may have prevented the need for data reduction (e.g. Latent Transition Analysis; repeated measures analysis). However, these methods would not have allowed us to explore individual-level trajectories, children with and without co-occurrence and the complexity of movement between ages 5 and 11, as they necessitate aggregation of data at the sample level.

### Comparison with other studies

Previous studies have explored co-occurrence cross-sectionally.[11,12] Our longitudinal findings concur with evidence from a study with US children, which suggests that co-occurrence is more common in boys and children from disadvantaged homes.[11] Extending the cross-sectional work, we showed that this social patterning is evident in trajectories of co-occurrence across childhood and early adolescence. Boys, children of mothers with lower educational attainment and those from lower income homes were more likely to have experience co-occurrence during this period of childhood. They were at greater risk of experiencing co-occurring health conditions at age 5, and were more likely to move into, out of co-occurrence, have fluctuating and constant co-occurrence up at age 11. This was true regardless of whether measures of socio-economic circumstances were recorded at 9 months, at primary school entry (age 5) or at school exit (age 11), suggesting that inequalities persist over time. This was also evidenced by similar findings regardless of whether children moved into or out of co-occurrence at age 7 or 11, i.e. it did not matter whether children were in co-occurrence for a longer or shorter period of time.

The equalization hypothesis suggests that such inequalities in health are reduced as children age, due to the greater influence of school and peers rather than the family and home environment.[14] Our work, which showed that more children moved into co-occurrence than moved out between age 5–11, and that there were socio-demographic differences in the trajectories *into* and *constant* co-occurrence, does not support this hypothesis. Although equalization has been shown to occur for individual health conditions, including two in our co-occurrence index (injuries and mental health),[14] we did not see dissipation of inequalities in co-occurrence as age increased. It may be that co-occurrence is akin to chronic illness (i.e. in terms of burden), where inequalities have not been shown to dissipate,[14] or that the children at age 11 are not yet old enough to exhibit equalization in health.[14] Children from lower income homes were however more likely to move out of co-occurrence here (in part due to having co-occurring conditions initially). This movement may relate to the specific health conditions a child has. Future work is required to establish whether this is the case, and determine if those with certain health conditions may be disproportionately affected by co-occurrence over time.

### Implications for policy and practice

Single conditions are generally used to measure the success of healthcare provision,[7,20] but this may not reflect the burden of ill-health experienced by a significant minority of children who have co-occurring conditions. Approximately 60% of children had relatively stable health in this sample, either with no co-occurrence (53%) or constant co-occurrence (7%). However, despite the promotion of optimal child health as a public health priority in the UK since 1997,[15–17] a sizeable group of children in this sample moved into, had fluctuating or constant co-occurrence. These children were more likely to be boys, and be from more disadvantaged homes, indicating inequalities in health burden exist across childhood.

A number of conditions have been shown to occur together previously, and may share common etiologies (e.g. obesity and lack of emotional wellbeing[8]). Yet despite this, we identified 32 different combinations of conditions co-occurring together at each age. Some likely share common etiological pathways (e.g. obesity and mental health) but the disease pathways for others (e.g. asthma, injury and obesity) remain to be determined. Our findings suggest that a child’s socio-economic circumstances may predict co-occurrence for a range of diverse pathways across childhood. However, we were not able to explore how other factors such as the role of cognition impact co-occurrence. Further research is therefore required to establish why certain sub-groups of children have a greater burden of co-occurrence across childhood, and to determine how services can better promote children’s health.[35,36]

## Supporting Information

S1 Table Prevalence of adverse health outcomes at ages 5, 7 and 11. (DOCX)

S2 Table Prevalence of co-occurring conditions at age 5, 7 and 11 (for the top 90% of conditions, due to small subsequent numbers). (DOCX)

S3 Table Imputed weighted associations between co-occurrence trajectories (5–11 years) and socio-demographic circumstances (n = 18 296). (DOCX)
