# Supplementary material for: Co-Occurrence of Health Conditions during Childhood: Longitudinal Findings from the UK Millennium Cohort Study (MCS)
Source: PLoS One. 2016 Jun 9;11(6):e0156868. doi: 10.1371/journal.pone.0156868 (PMC4900599; doi:10.1371/journal.pone.0156868)
Supplement: S1 Table — (DOCX) [file pone.0156868.s001.docx]

**S1 Table Prevalence of adverse health outcomes at ages 5, 7 and 11**

|  |  |  | % Prevalence (n) |  |
| --- | --- | --- | --- | --- |
| Number of outcomes | | Age 5 | Age 7 | Age 11 |
| No co-occurrence | 0 | 37.3 (3595) | 42.7 (4151) | 30.4 (2982) |
|  | 1 | 37.8 (3642) | 37.6 (3601) | 39.6 (3792) |
| Co-occurrence | 2 | 18.5 (1729) | 15.1 (1394) | 21.0 (1955) |
|  | 3/+ | 6.4 (582) | 4.6 (402) | 9.1 (819) |
