# Supplementary material for: Co-Occurrence of Health Conditions during Childhood: Longitudinal Findings from the UK Millennium Cohort Study (MCS)
Source: PLoS One. 2016 Jun 9;11(6):e0156868. doi: 10.1371/journal.pone.0156868 (PMC4900599; doi:10.1371/journal.pone.0156868)
Supplement: S2 Table — (DOCX) [file pone.0156868.s002.docx]

**S2 Table Prevalence of co-occurring conditions at age 5, 7 and 11 (for the top 90% of conditions, due to small subsequent numbers)**

| **Health conditions (Age 5)** | **N** | **%** | **Health conditions (Age 7)** | **N** | **%** | **Health conditions (Age 11)** | **N** | **%** |
| --- | --- | --- | --- | --- | --- | --- | --- | --- |
| No outcome | 3595 | 37.7 | No outcome | 4151 | 43.5 | No outcome | 2982 | 31.2 |
| Injury | 1242 | 13 | Problem Weight | 1311 | 13.7 | Injury | 1633 | 17.1 |
| Problem Weight | 1120 | 11.7 | Injury | 1193 | 12.5 | Problem Weight | 1412 | 14.8 |
| Wheeze | 590 | 6.2 | Wheeze | 509 | 5.3 | Injury / Problem Weight | 745 | 7.8 |
| Longstanding illness | 417 | 4.4 | Socio-emotional difficulties | 382 | 4.0 | Socio-emotional difficulties | 312 | 3.3 |
| Injury / Problem Weight | 397 | 4.2 | Injury / Problem Weight | 361 | 3.8 | Wheeze | 227 | 3.4 |
| Socio-emotional difficulties | 273 | 2.9 | Longstanding illness | 206 | 2.2 | Longstanding illness | 208 | 2.2 |
| Wheeze / Injury | 225 | 2.4 | Wheeze / Problem Weight | 202 | 2.1 | Injury / Socio-emotional difficulties | 189 | 2.0 |
| Wheeze / Problem Weight | 208 | 2.2 | Wheeze / Injury | 170 | 1.8 | Socio-emotional difficulties / Problem Weight | 187 | 2.0 |
| Injury / Longstanding illness | 195 | 2.0 | Socio-emotional difficulties / Problem Weight | 158 | 1.7 | Wheeze / Injury | 173 | 1.8 |
| Wheeze / Longstanding illness | 155 | 1.6 |  |  |  | Injury / Longstanding illness | 152 | 1.6 |
| Longstanding illness / Problem Weight | 153 | 1.6 |  |  |  | Injury / Socio-emotional difficulties / Problem Weight | 123 | 1.3 |
| Injury / Socio-emotional difficulties | 121 | 1.3 |  |  |  | Wheeze / Problem Weight | 123 | 1.3 |
|  |  |  |  |  |  | Longstanding illness / Problem Weight | 120 | 1.3 |
|  |  |  |  |  |  | Wheeze / Longstanding illness | 119 | 1.3 |
|  |  |  |  |  |  |  |  |  |
