# Supplementary material for: Co-Occurrence of Health Conditions during Childhood: Longitudinal Findings from the UK Millennium Cohort Study (MCS)
Source: PLoS One. 2016 Jun 9;11(6):e0156868. doi: 10.1371/journal.pone.0156868 (PMC4900599; doi:10.1371/journal.pone.0156868)
Supplement: S3 Table — (DOCX) [file pone.0156868.s003.docx]

**S3 Table Imputed weighted associations between co-occurrence trajectories (5-11 years) and socio-demographic circumstances**

**(n=18 296)**

|  | **Adjusted RRR (95% CI)** | | | | |
| --- | --- | --- | --- | --- | --- |
|  | **No co-occurrence** | **Into co-occurrence** | **Out of co-occurrence** | **Fluctuating co-occurrence** | **Constant co-occurrence** |
| **Sex** (ref: Male) |  |  |  |  |  |
| Female | 1.00 | **0.82 (0.73, 0.93)** | **0.82 (0.72, 0.93)** | **0.72 (0.61, 0.86)** | **0.60 (0.51, 0.71)** |
| **Ethnicity** (ref: White) |  |  |  |  |  |
| Mixed | 1.00 | 1.04 (0.66, 1.51) | **1.53 (1.03, 2.27)** | 1.00 (0.58, 1.70) | 1.36 (0.87, 2.13) |
| Indian | 1.00 | 0.65 (0.45, 1.09) | 1.12 (0.74, 1.70) | 0.92 (0.55, 1.56) | 0.93 (0.43, 2.02) |
| Pakistani/ Bangladeshi | 1.00 | **0.65 (0.48, 0.88)** | **1.38 (1.08, 1.75)** | 0.89 (0.62, 1.28) | **0.65 (0.46, 0.92)** |
| Black | 1.00 | **0.59 (0.40, 0.86)** | 1.05 (0.69, 1.62) | 0.91 (0.56, 1.49) | 0.53 (0.27, 1.05) |
| Other | 1.00 | 0.63 (0.32, 1.25) | 0.99 (0.55, 1.79) | 0.64 (0.29, 1.41) | 0.53 (0.24, 1.17) |
| **Maternal Educational attainment*** (ref: Higher/degree) |  |  |  |  |  |
| Diploma | 1.00 | 1.09 (0.87, 1.37) | 0.88 (0.62, 1.24) | 1.24 (0.94, 1.65) | 1.44 (0.92, 2.24) |
| A-levels | 1.00 | 1.03 (0.86, 1.35) | 0.98 (0.73, 1.31) | 1.23 (0.89, 1.69) | 1.38 (0.89, 2.15) |
| GCSEs | 1.00 | **1.22 (1.02, 1.47)** | **1.22 (0.99, 1.50)** | 1.24 (0.95, 1.60) | **2.00 (1.44, 2.77)** |
| None | 1.00 | **1.29 (1.02, 1.64)** | **1.58 (1.21, 2.07)** | **1.60 (1.16, 2.20)** | **2.50 (1.78 3.51)** |
| **Household income*** (ref: Q1 High) |  |  |  |  |  |
| Q2 | 1.00 | 1.18 (0.97, 1.43) | **1.36 (1.11, 1.66)** | 1.02 (0.77, 1.36) | 1.19 (0.83, 1.72) |
| Q3 | 1.00 | **1.29 (1.06, 1.57)** | **1.43 (1.13, 1.81)** | 1.25 (0.93, 1.69) | **1.85 (1.29, 2.64)** |
| Q4 | 1.00 | **1.50 (1.21, 1.86)** | **1.52 (1.21, 1.89)** | **1.46 (1.13, 1.89)** | **2.46 (1.75, 3.45)** |
| Q5 (low) | 1.00 | **1.60 (1.30, 1.97)** | **1.85 (1.44, 2.36)** | **1.63 (1.22, 2.18)** | **3.55 (2.50, 5.03)** |

* Measured at age 9 months
